# A Lectin-EGF antibody promotes regulatory T cells and attenuates nephrotoxic nephritis via DC-SIGN on dendritic cells

**DOI:** 10.1186/1479-5876-11-103

**Published:** 2013-04-29

**Authors:** Minchao Cai, Jing Wu, Chaoming Mao, Jianmin Ren, Pu Li, Xiao Li, Jiuchang Zhong, Chundi Xu, Tong Zhou

**Affiliations:** 1 Department of Pediatrics, Ruijin Hospital, Shanghai Jiao Tong University School of Medicine, 197 Ruijin Er Road, Shanghai 200025, China; 2 Department of Nephrology, Ruijin Hospital, Shanghai Jiao Tong University School of Medicine, 197 Ruijin Er Road, Shanghai 200025, China; 3 Shanghai Institute of Immunology and Institute of Health Sciences, Shanghai Jiao Tong University School of Medicine, 227 South Chongqing Road, Shanghai, 200025, China; 4 Department of Nuclear Medicine, The Affiliated Hospital of Jiangsu University, 438 Jiefang Road, Zhenjiang, 212001, China; 5 State Key Laboratory of Medical Genomics and Shanghai Institute of Hypertension, Ruijin Hospital, Shanghai Jiaotong University School of Medicine, 197 Ruijin Er Road, Shanghai, 200025, China

**Keywords:** DC-SIGN, Dendritic cells, Regulatory T cells, Glomerulonephritis

## Abstract

**Background:**

Interactions between dendritic cells (DCs) and T cells play a critical role in the development of glomerulonephritis, which is a common cause of chronic kidney disease. DC-specific intercellular adhesion molecule-3-grabbing non-integrin (DC-SIGN), an immune-regulating molecule of the C-type lectin family, is mainly expressed on DCs and mediates DC adhesion and migration, inflammation, activation of primary T cells. DC-SIGN triggers immune responses and is involved in the immune escape of pathogens and tumours. In addition, ligation of DC-SIGN on DCs actively primes DCs to induce Tregs. Under certain conditions, DC-SIGN signalling may result in inhibition of DC maturation, by promoting regulatory T cell (Treg) function and affecting Th1/Th2 bias.

**Methods:**

A rat model of nephrotoxic nephritis was used to investigate the therapeutic effects of an anti-lectin-epidermal growth factor (EGF) antibody on glomerulonephritis. DCs were induced by human peripheral blood mononuclear cells *in vitro*. The expression of DC surface antigens were detected using flow cytometry; the levels of cytokines were detected by ELISA and qPCR, respectively; the capability of DCs to stimulate T cell proliferation was examined by mixed lymphocyte reaction; PsL-EGFmAb targeting to DC-SIGN on DCs was identified by immunoprecipitation.

**Results:**

Anti-Lectin-EGF antibody significantly reduced global crescent formation, tubulointerstitial injury and improved renal function impairment through inhibiting DC maturation and modulating Foxp3 expression and the Th1/Th2 cytokine balance in kidney. Binding of anti-Lectin-EGF antibody to DC-SIGN on human DCs inhibited DC maturation, increased IL-10 production from DCs and enhanced CD4^+^CD25^+^ Treg functions.

**Conclusions:**

Our results suggest that treatment with anti-Lectin-EGF antibody modulates DCs to suppressive DCs and enhances Treg functions, contributing to the attenuation of renal injury in a rat model of nephrotoxic nephritis.

## Background

Glomerulonephritis (GN) is a common cause of renal failure. Tubulointerstitial inflammation is a major contributor of GN progression to renal failure, even in crescentic GN with severe glomerular injury [1,2]. Evidences suggest that crescent formation is driven by Th1-dominated nephritogenic immune responses [3-6]. Development of Th1 or Th2 immunity is controlled by dendritic cells (DCs). In different forms of GN, renal function and prognosis closely correlate with the extent of DC infiltration into the tubulointerstitium [7-9].

DCs are critical in the control of immune responses and homoeostasis. Mature DCs induce Th1, Th2 and Th17 effector T-cells, whereas immature DCs drive the development of regulatory T cells (Tregs), which maintain tolerance to self-antigens and inhibit excessive immune responses by producing IL-10 and TGF-β [9-14]. DC maturation is pathogen-specific and exhibits a large degree of plasticity [15]. The nature of the antigen determines the balance towards immunity or tolerance. DCs express a large array of cell surface lectins and lectin-like molecules which are receptors for cognate antigens [16-20]. DC-specific intercellular adhesion molecule-3-grabbing non-integrin (DC-SIGN), a member of the C-type lectin family, mediates DC adhesion and migration, inflammation, activation of primary T cells, contributes to immune responses and the immune escape of pathogens and tumors [21-23]. DC-SIGN is abundantly expressed on immature DCs (iDCs) and is down-regulated during maturation [21]. DC-SIGN signaling can induce opposite immune responses [18,22,24]. Ligation of DC-SIGN on DCs actively primes DCs to induce Tregs [25]. Thus, targeting DC-SIGN may be a useful strategy to suppress inflammatory responses, which could be beneficial in managing autoimmunity.

Previously we demonstrated that DCs treated *in vitro* with an anti-lectin-EGF domain monoclonal antibody originally developed against P-selectin (PsL-EGFmAb) displayed low expression of co-stimulatory molecules and an impaired capability to stimulate CD4^+^ T cells [26], indicating suppression of DC maturation and function. However, the underlying mechanism remains unclear. Thus, we administered PsL-EGFmAb to a rat model of nephrotoxic nephritis (NTN), an immune-mediated animal model of human crescentic GN, to investigate whether PsL-EGFmAb could affect DC maturation and Treg- and Th1/Th2-related gene expression in renal tissues, and further investigate the mechanism by which this antibody affects DCs *in vitro*. We infer that PsL-EGFmAb might interact with DC-SIGN that also contains a lectin domain to inhibit DC maturation and induce Tregs that inhibit effector T cell functions. Our results showed that a lectin-EGF antibody targeting DC-SIGN on dendritic cells promotes regulatory T cells and attenuates nephrotoxic nephritis.

## Methods

### Animals and treatment protocol

Male 6-8-week old Wistar-Kyoto rats were bred and kept in specific-pathogen-free conditions in an animal facility. Rabbit nephrotoxic serum was generated as described previously [27,28]. The amount of nephrotoxic serum per gram of body weight used in this study (2.0 or 2.5 mg immunoglobulin per gram body weight) was within the linear range of a dose response. Eighteen rats were equally and randomly assigned to three groups and received different injection by tail vein: control group rats injected with normal rat serum; NTN group rats injected with two doses of mouse IgG (2 μg per gram of rat body weight) at 0 and 2 h after nephrotoxic serum injection; PsL-EGFmAb-treated group rats received two injections of PsL-EGFmAb, a mouse anti-human monoclonal antibody established in our lab (2 μg per gram of rat body weight) at 0 and 2 h after nephrotoxic serum injection. On day 14, the rats were anesthetized with ketamine and sacrificed. Rats in every group were placed in metabolic cages to collect 24 hours urine and detect urine creatinine. Blood was got from inner canthal vein of rats in every group. Serum creatintine (Scr) and urea nitrogen were detected by automatic biochemical analyzer. Creatinine clearance rate (CCr) was got by the formula according to creatintine and urine output in serum and urine. The kidneys were quickly isolated and fixed in 10% buffered formaldehyde. The study was approved by the Ethics Committee of Ruijin Hospital, Shanghai Jiaotong University School of Medicine.

### Renal function studies

For urine sample collection, rats were housed in metabolic cages. Albuminuria was determined by standard ELISA analysis. Blood samples for blood urea nitrogen (BUN) measurement were obtained at the time of sacrifice. Scr and BUN were measured by standard laboratory methods. Ccr (ml/min) was used to estimate glomerular filtration rate (GFR).

### Periodic Acid Schiff staining

To evaluate glomerular and tubulointerstitial injury, formalin-fixed rat renal tissue was embedded in paraffin, sectioned at 4 μm, and stained with Periodic Acid Schiff (PAS) for histological analysis. Tubulointerstitial lesions were scored as follows: 0, no tubular damage, no interstitial edema; 0.5, thinning of the brush borders; 1, thinning of the tubular epithelial; 2, denudation of the tubular basement membrane; 3, tubular necrosis.

### Analysis of MHC class-II, DC-SIGN and CD80 expressed on renal DCs

DCs were isolated from rat kidneys as previously described [29]. Briefly, kidneys were finely minced and digested for 45 min at 37°C with 2 mg/ml collagenase D in RPMI 1640 medium with 10% heat-inactivated fetal calf serum. Cell suspensions were filtered through 30 μm nylon mesh, and washed with HBSS without Ca^2+^ and Mg^2+^ containing 10 mmol/L EDTA, 0.1% BSA and 10 mM Hepes. Density centrifugation was performed at 1700 × *g* for 20 min at 4°C using 1.080 g/ml of Nycodenz (Axis-Shield). The interphase cells were harvested and isolated with anti-rat OX62 micro-beads (Miltenyi Biotec, Bergisch Gladbach, Germany). Isolated cells (5 × 10^5^) were stained with FITC- and PE-labeled mAbs specific for MHC class-II and CD80. In addition, 1 × 10^5^ DCs were stained indirectly with DC-SIGN using goat anti-rat DC-SIGN polyclonal antibody and PE-conjugated donkey anti-goat IgG mAb. Phenotypic analysis was performed by flow cytometry using a FACS Calibur (BD FACSAria™ Cell Sorter).

### Real-time PCR assays

Renal tissue was lysed and total RNA extracted using Trizol reagent (Invitrogen, Carlsbad, CA, USA). cDNA was synthesized using the RevertAid First Strand cDNA Synthesis Kit (Fermentas, Burlington, Canada), following the manufacturer’s recommendations. The cycle number at which the fluorescence increased above the threshold was designated as the threshold cycle (CT). Primer specificity was assessed by melting curve. These samples were then analyzed for the expression of IFN-γ, TNF-α, IL-6, IL-4, Foxp3, IL-10, TGF-β, and GAPDH genes by PCR using the SYBR GREEN PCR Master Mix (Applied Biosystems, Carlsbad, CA, USA) and the ABI PRISM 7700 Sequence Detection System. The sequences of the specific primer pairs used in each case were listed in Table 1. PCR was performed three times as follows: 40 cycles of denaturing at 95°C for 15 s and annealing/extension at 60°C for 1 min. Results were normalized to GAPDH expression using the 2^–ΔΔCt^ method.

**Table 1:** Sequences of specific primer pairs

	**Forward**	**Reverse**
IFN-γ	5′-AACCAGGCCATCAGCAACAAC A -3′	5′-ACCGACTCCTTTTCCGCTTCCT-3′
TNF-α	5′-GGTGATCGGTCCCAACAAGGA -3′	5′-CACGCTGGCTCAGCCACTC-3′
IL-6	5′-ATATGTTCTCAGGGAGATCTTGGA A-3′	5′-GTGCATCATCGCTGTTCATACA-3′
IL-4	5′-AACACCACGGAGAACGAGCTC ATC-3′	5′- AGTGAGTTCAGACCGCTGACACCT -3′
Foxp3	5′-CCCAGGAAAGACAGCAACCTT -3′	5′- CTGCTTGGCAGTGCTTGAGAA -3′
IL-10	5′-GCCAAGCCTTGTCAGAAATGA -3′	5′- TTTCTGGGCCATGGTTCTCT -3′
TGF-β	5′-ACCGGGTGGCAGGCGAGAG -3′	5′- CGGGACAGCAATGGGGGTTCT -3′
GAPDH	5′-AGGACCAGGTTGTCTCCTGT -3′	5′- TTACTCCTTGGAGGCCATGT -3′

### Cell isolation

PBMCs were obtained from whole blood of healthy donors by Ficoll density gradient centrifugation (Sigma-Aldrich, St. Louis, MO, USA). Then, monocytes were isolated by positive selection with human anti-CD14 microbeads (Miltenyi Biotec), following the manufacturer’s instructions. To generate imDCs, isolated CD14^+^ monocytes (5 × 10^5^/ml) were incubated at 37°C for 5–7 days in RPMI 1640 complete medium (Invitrogen), containing 10% fetal calf serum and supplemented with 50 ng/ml human GM-CSF and 20 ng/ml human IL-4 (R&D Systems). To obtain mDCs, imDCs were incubated for 48 h in the presence of 50 ng/ml TNF-α (R&D Systems). PsL-EGFmAb (10 μg/ml) was added to the culture 2 h before TNF-α addition to obtain PsL-EGFmAb-treated DCs. The expression of HLA-DR, CD80, CD86, CD83 and DC-SIGN on the surface of DCs was confirmed by flow cytometry analysis.

Human CD4^+^ T cells were isolated from PBMCs by negative selection and the fraction of remaining cells were used to further isolate CD4^+^CD25^-^ T and CD4^+^CD25^+^ T cells by negative and positive selection using a human CD4^+^CD25^+^ Treg isolation kit (Miltenyi Biotec), following the manufacturer's instructions.

### Flow cytometry analysis

Expressions of surface antigens on DCs (1 × 10^5^) were assessed with the following mAbs: anti-human HLA-DR and CD83, PE-labeled anti-rat CD80, anti-human CD80 and DC-SIGN, allophycocyanin-labeled anti-human CD86, goat anti-rat DC-SIGN antibody and PE-conjugated donkey anti-goat IgG. Appropriate isotype antibodies were used as controls. The proportion of CD4^+^CD25^+^Foxp3^+^ T cells in the CD4^+^ T cell population was determined using a human Treg staining kit (eBioscience), according to manufacturer’s instructions. In brief, after CD4 and CD25 surface staining, cells (5 × 10^5^) were washed and fixed at 4°C for 60 min in the dark using fixation/permeabilization solution. Cells were then stained intracellularly for Foxp3.

To investigate whether PsL-EGFmAb can bind to DC-SIGN, flow cytometry analysis was performed. First, we used PE-labeled anti-DC-SIGN mAb and PsL-EGFmAb together with FITC-labeled anti-mouse IgG mAb to detect expression of DC-SIGN on imDCs (1 × 10^5^) separately. Then imDCs were incubated with goat anti-human DC-SIGN antibody for 2 h before detection of DC-SIGN expression by PsL-EGFmAb together with FITC-labeled anti-mouse IgG mAb. The percentage of positive cells was analyzed in a FACSCalibur flow cytometer (BD Biosciences), using Flowjo software (v. 5.7.2, Tree Star Inc., Ashland, OR, USA).

### Cytokine assays

Levels of IFN-γ, IL-12, TGF-β, IL-10 and IL-6 in cell cultures were determined by ELISA (Biosource, Carlsbad, CA, USA). Briefly, cell culture supernatants were collected and the cytokine concentration was analyzed by a specific solid phase sandwich enzyme immunoassay, following the manufacturer’s instructions.

### Allogeneic mixed cell proliferation assays

The ability of DCs to stimulate CD4^+^ T cells was assayed by mixed lymphocyte reaction. Allogeneic CD4^+^ T cells (2 × 10^5^) isolated from rat peripheral blood mononuclear cells by magnetic bead-labeled anti-rat CD4 mAb (Miltenyi Biotec) were incubated with irradiated (30 Gy) DCs (2 × 10^4^) isolated from rat kidneys in control, NTN and PsL-EGFmAb-treated groups with anti-rat OX62 micro-beads as mentioned above at a 10:1 ratio in a 96-well U-bottomed plate at 37°C for five days. T cell proliferation was assessed after five days of co-culture by [methyl-^3^H]thymidine ([^3^H]TdR, 5.0 μCi/ml) incorporation in a 16-h pulse. For this purpose, cells were harvested with a semi automated device, and the incorporation of [^3^H]TdR was determined in a liquid scintillation counter. Triplicate wells were cultured for each group.

To analyze the stimulatory potential of human DCs on allogeneic CD4^+^ T, CD4^+^CD25^+^ T and CD4^+^CD25^-^ T cells, T cells (2 × 10^5^) were co-cultured for five days with imDCs, mDCs or PsL-EGFmAb-treated DCs (2 × 10^4^). T cells without DC or PsL-EGFmAb treatment were used as a control. T cell proliferation was assessed as mentioned above. All of these experiments were conducted in triplicate.

### Suppression assays

To analyze the suppressive function of the Tregs induced *in vitro* by DCs, autologous mixed cell cultures were performed [30]. Briefly, CD4^+^CD25^+^ T cells recovered after five days of co-culture with DCs (T1 cells), were maintained in culture for two additional days in the presence of IL-2 (50 U/ml) (R&D Systems). T1 cells or MACS freshly isolated normal human CD4^+^CD25^+^ Tregs (2 × 10^5^) were mixed with autologous CD4^+^ CD25^-^ T cells (1 × 10^5^) and stimulated with anti-CD3 (5 μg/ml) mAb plus anti-CD28 (1 μg/ml) mAb. After two days of cell culture, 5.0 μCi/ml [^3^H]TdR was added and the cells were harvested 16 h later. Results were expressed as fold increases in [^3^H]TdR incorporation.

### Neutralization assays

To determine which cytokines secreted by PsL-EGFmAb-treated DCs were involved in CD4^+^CD25^+^ Treg priming, anti-IL-6 (5 μg/ml), anti-IL-10 (10 μg/ml), anti-TGF-β (10 μg/ml), anti-IL-4 (10 μg/ml), anti-TNF-α (5 μg/ml), anti-IL-12 (10 μg/ml) and anti-IFN-γ (5 μg/ml) mAbs were used. PsL-EGFmAb-treated DCs (2 × 10^4^) were co-cultured with MACS freshly isolated human CD4^+^CD25^+^ T cells (2 × 10^5^) for five days in the presence of the different neutralizing antibodies mentioned above. Mouse IgG was also added as an isotype control. CD4^+^CD25^+^ T cells alone were used as a negative control. T cell proliferation was assessed by [^3^H]TdR (5.0 μCi/ml) incorporation in a 16-h pulse. Cells were harvested with a semi automated device, and the incorporation of [^3^H]TdR was determined in a liquid scintillation counter.

Next, to determine which cytokines secreted by T1 cells inhibited CD4^+^CD25^-^ effector T cell proliferation, the same neutralizing antibodies at similar doses were used. PsL-EGFmAb-treated DC-induced T1 cells (2 × 10^5^) were co-cultured with 1 × 10^5^ MACS freshly isolated human CD4^+^CD25^-^ T cells for five days in the presence of different neutralizing antibodies. Mouse IgG was also added as an isotype control, while CD4^+^CD25^-^ T cells alone were used as a negative control. T cell proliferation was assessed using the method mentioned above. All of these experiments were conducted in triplicate and the results were expressed as fold increases in [^3^H]TdR incorporation.

### siRNA Sequences (DC-SIGN)

Sequences of various siRNAs used are as follows: DC-SIGN antisense, 5′-ATT TGT CGT CGT TCC AGC CAT-3′; DC-SIGN sense, 5′-ATG GCT GGA ACG ACA CAA A-3′; DC-SIGN antisense scrambled control, 5′-CAC ACC ACA TCT TTC CGT CAC-3′; DC-SIGN sense scrambled control, 5′-GTG ACG GAA AGA TGT GGT G-3′. RNA oligonucleotides were custom synthesized by Dharmacon Research Inc (Lafayette, CO, USA) with an overhang of 2 thymidine residues (dTdT) at the 3′ end. The RNA oligonucleotides were dissolved in Tris-EDTA (10 mM Tris–HCl, pH 8.0; and 1 mM EDTA) as 200 μM solutions and were stored at -20°C. Double-stranded siRNA molecules were generated by mixing the corresponding pair of sense and antisense RNA oligonucleotides in annealing buffer (30 mM HEPES-KOH, pH 7.9; 100 mM potassium acetate; and 2 mM magnesium acetate) at 20 μM and then by incubating the reaction mixture at 95°C for 2 min, followed by gradual cooling to room temperature. The siRNAs were then aliquoted and stored at -20°C.

### Transfection of siRNAs

Twenty-four-hours before siRNA transfection, DCs (1 × 10^6^) were seeded in 6-well plates in OPTI-minimal essentials medium (OptiMEM; Invitrogen, Grand Island, NY, USA) containing 10% fetal bovine serum (FBS) with no antibiotics. Cells were seeded per 6-well plate to give 30% to 50% confluency at the time of the transfection. The siRNAs were transfected at a final concentration of 50 nM using Oligofectamine (Invitrogen) according to the manufacturer’s recommendations. The control (untransfected) cells received either Oligofectamine alone or Oligofectamine plus scrambled sequence. The siRNAs added to control cells were incubated for 24–48 and 72 hours and the cells were harvested and RNA was extracted.

### Immunoprecipitation

ImDCs (5 × 10^5^) were lysed in lysis buffer (1% Triton-X-100, 10 mM TEA pH 8.2, 150 mM NaCl, 1 mM MgCl_2_ and 1 mM CaCl_2_) containing a cocktail of EDTA-free protease inhibitors for 1 h at 4°C. Cell lysates were immunoprecipitated with PsL-EGFmAb-coated or mouse IgG-coated Agarose A/G beads (Pierce, Appleton, WI, USA), following the manufacturer's instructions. Briefly, cell lysates were incubated with PsL-EGFmAb-coated or mouse IgG-coated beads overnight at 4°C on an orbital shaker. The beads were then collected and washed three times with 800 μl of ice-cold PBS followed by pulse centrifugation. After washing, beads were resuspended in 60 μl of 2 × sample buffer and gently mixed. DC-SIGN was detected by western blotting using the protocol mentioned above. Cell lysates without immunoprecipitation were used as positive controls.

### Statistical analysis

SPSS software, version 11.0, was used for statistical analyses. Data are presented as the mean ± SD. Statistically significant differences between groups were determined using the Student’s *t* test or Mann–Whitney *U-*test, as appropriate. A value of *p* < 0.05 was considered significant.

## Results

### PsL-EGFmAb treatment attenuates renal lesions and improves renal function in a rat model of NTN

**Figure 1:**
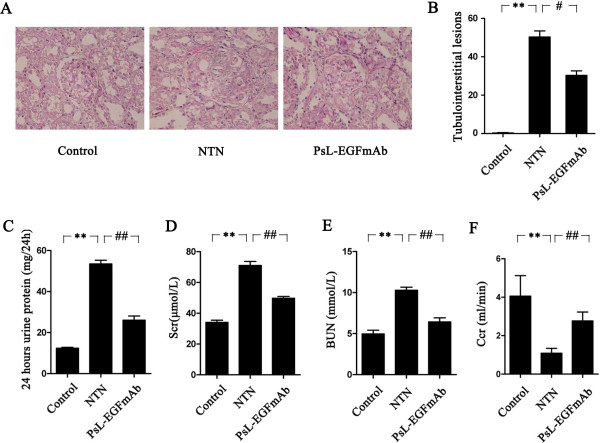
**Effect of PsL-EGFmAb on renal pathology and function. A**, PAS staining of renal tissues and corresponding quantification (final magnification × 200). Renal tissues were harvested on day 14. **B**, Tubulointerstitial damage was evaluated according to the scoring system we mentioned in the Methods. **C-F**, Twenty-four-hour urine proteins (**C**), Scr (**D**) and BUN (**E**) of nephritic rats were significantly elevated, whereas CCr (**F**) levels were significantly reduced on day 14 compared with non-nephritic controls (*p* < 0.01). PsL-EGFmAb treatment significantly reduced twenty-four-hour urine proteins (**C**), Scr (**D**), BUN (**E**) levels and increased CCr (**F**) compared with NTN group (*p* < 0.01), indicating improvement of renal function after PsL-EGFmAb treatment. The mean ± SD of three independent experiments is shown. **p < 0.01 *vs.* control; ^#^*p* < 0.05, ^##^*p* < 0.01 *vs. *NTN group.

A rat model of NTN was established and used to test the therapeutic effect of PsL-EGFmAb [31]. Rats injected with nephrotoxic serum developed typical NTN inflammation on day 14 after induction, characterized by enlarged glomeruli, global cellular crescent formation in more than 50% of glomeruli (59.41 ± 2.22%, *p* < 0.01) destruction of Bowman's capsule wall, abruption of the basement membranes of glomeruli and tubules, interstitial edema and diffuse inflammatory cell infiltration (Figure 1A). Compared to control rats injected with IgG, rats treated with PsL-EGFmAb showed attenuated glomerular and tubulointerstitial lesions (Figure 1A and 1B). The proportion of glomeruli with crescent formation was significantly reduced (33.90 ± 6.46%, *p* < 0.01).

Twenty-four-hour urinary proteins (Figure 1C), Scr (Figure 1D) and BUN (Figure 1E) of nephritic rats were significantly elevated, and Ccr (Figure 1F) was significantly decreased on day 14 compared with non-nephritic controls (*p* < 0.01). In the PsL-EGFmAb-treated group, we observed a significant reduction in levels of urinary 24-hour proteins, BUN and Scr, and increased Ccr levels compared with the NTN group (*p* < 0.01), indicating that PsL-EGFmAb treatment improved renal function.

### PsL-EGFmAb treatment inhibits renal DC maturation

**Figure 2:**
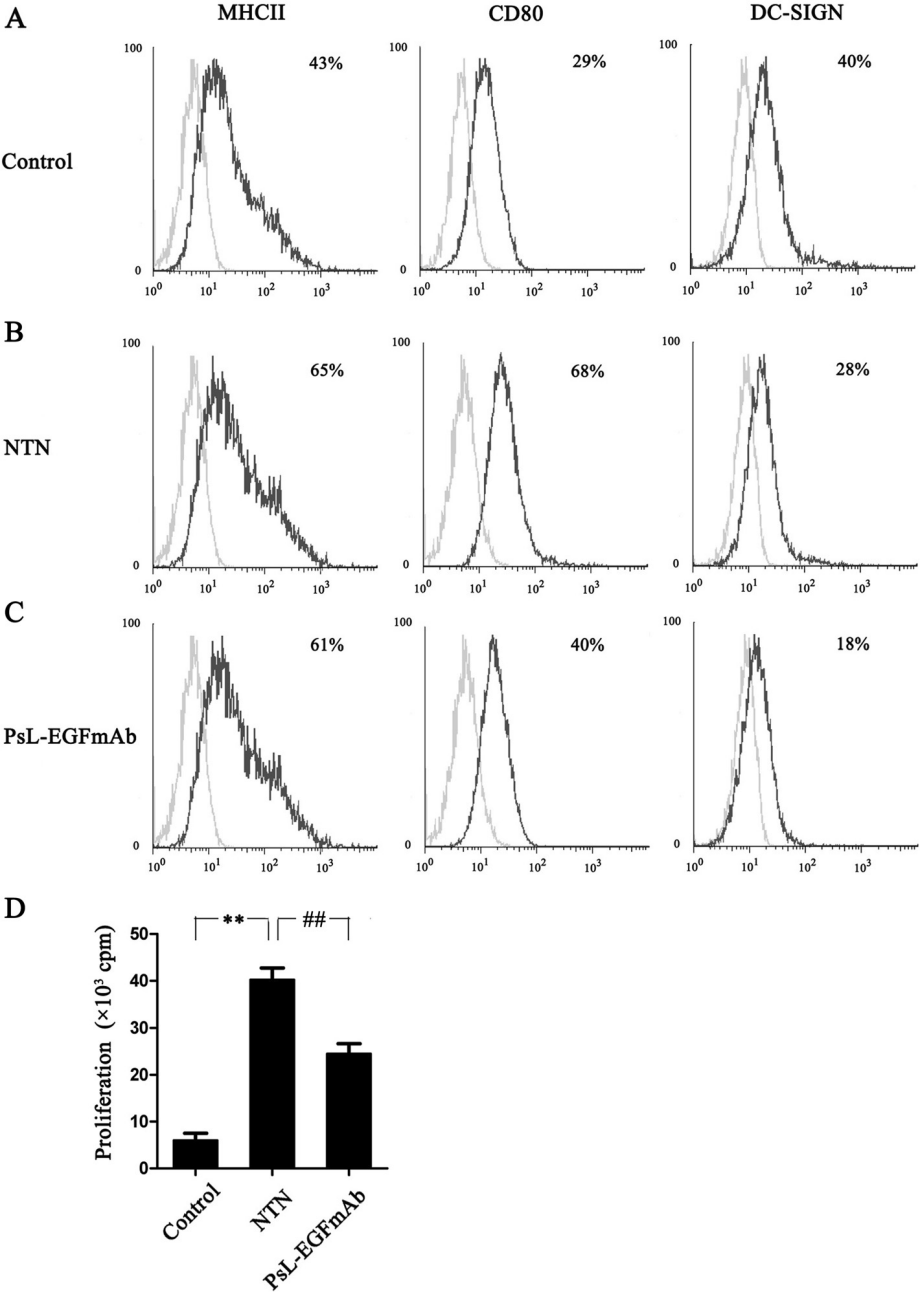
**Effect of PsL-EGFmAb on renal DC maturation. A**-**C**, Renal DCs from control (**A**), NTN (**B**) and PsL-EGFmAb (**C**) groups were freshly isolated by MACS using anti-rat OX62 micro-beads on day 14 and maturity of DCs was determined by flow cytometry detected by cell surface expression of MHC class-II, CD80 and DC-SIGN (black line). The isotype-matched controls are shown as gray line. **D**, Allogeneic mixed lymphocyte reactions were used to evaluate the proliferation of CD4^+ ^T cells induced by isolated DCs. MACS-isolated and irradiated DCs from rat kidneys of control, NTN and PsL-EGFmAb-treated groups were co-cultured with CD4^+ ^T cells from healthy rats at a 1:10 ratio for five days. T cell proliferation was assessed by [^3^H]TdR incorporation. The results were shown as fold increases in [^3^H]TdR incorporation. The mean ± SD of three independent experiments is shown. **p < 0.01 *vs. *control; ^##^*p* < 0.01 *vs. *NTN group.

To investigate whether treatment of PsL-EGFmAb could inhibit DC maturation *in vivo*, renal DCs were freshly isolated at two weeks. Injection of rats with rabbit nephrotoxic serum significantly up-regulated MHC class-II and CD80 expression but down-regulated DC-SIGN expression on renal DCs compared with controls. Treatment with PsL-EGFmAb significantly down-regulated DC-SIGN and CD80 expression and slightly decreased MHC class-II expression on DCs compared with controls treated with non-specific IgG. DC-SIGN expression was decreased after treatment with PsL-EGFmAb (Figure 2, A-C). The ability of DCs from the rat NTN group to stimulate T cells significantly improved but was reduced in the PsL-EGFmAb-treated group (Figure 2D). This suggests that PsL-EGFmAb treatment significantly inhibited DC-SIGN expression and DC maturation in the rat NTN group.

### PsL-EGFmAb treatment inhibits inflammatory cytokine expression, shifts the Th1/Th2 cytokine profile and up-regulates Treg-related gene expression in renal tissues

**Figure 3:**
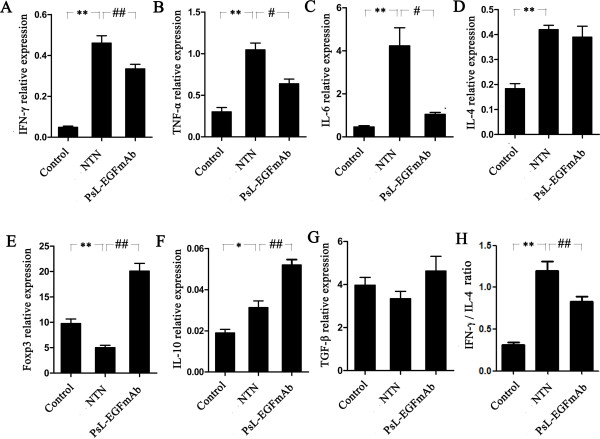
**The mRNA expression of cytokines in renal tissues. **Real-time PCR for IFN-γ (**A**), TNF-α (**B**), IL-6 (**C**), IL-4 (**D**), Foxp3 (**E**), IL-10 (**F**), TGF-β (**G**) mRNA expression and IFN-γ/ IL-4 ratio (**H**) in renal tissues. Expression of the genes of interest was normalized to that of the GAPDH gene, and described as 2^–ΔΔCt^. The mean ± SD of four independent experiments is shown. *p < 0.05, **p < 0.01 *vs. *control; ^#^*p* < 0.05, ^##^*p* < 0.01 *vs. *NTN group.

We measured the expression of three pro-inflammatory cytokines, interferon (IFN)-γ (Figure 3A), tumor necrosis factor (TNF)-α (Figure 3B) and interleukin (IL)-6 (Figure 3C) in renal tissues by real-time PCR. Administration of rabbit nephrotoxic serum to rats induced significant expression of all three cytokines compared with controls (*p* < 0.05). Interestingly, the expression of all three cytokines was significantly decreased in rats treated with PsL-EGFmAb compared rats from the NTN group (*p* < 0.05), indicating that PsL-EGFmAb elicits an anti-inflammatory effect in the rat NTN model.

We next investigated the Th1/Th2 cytokine profile. Non-immunized rats displayed a Th2-dominated cytokine profile characterized by higher levels of IL-4 (Figure 3D) compared to IFN-γ. Although injecting rats with rabbit nephrotoxic serum induced significant IL-4 and IFN-γ secretion (*p* < 0.05), the increase in IFN-γ expression was much higher than IL-4, indicating a switch from a Th2- to a Th1-dominated profile (Figure 3A, D, H). Interestingly, PsL-EGFmAb treatment not only decreased IFN-γ expression, but also reversed the IFN-γ/IL-4 ratio compared with the rat NTN group (Figure 3A, D, *p* < 0.05).

It is well established that Tregs can inhibit both Th1 and Th2 type immune responses. To further explore the cause of decreased levels of inflammatory cytokines after PsL-EGFmAb treatment, we investigated the expression of Treg-related factors such as Foxp3, IL-10 and TGF-β in renal tissues. There was a significant down-regulation of Foxp3 expression (Figure 3E) after rats were injected with the rabbit nephrotoxic serum (*p* < 0.05), whereas PsL-EGFmAb treatment significantly up-regulated Foxp3 expression in both the NTN group and non-immunized rats (*p* < 0.05), indicating the possible up-regulation of Tregs after PsL-EGFmAb treatment. We then examined expression of IL-10 and TGF-β, the two main cytokines promoting Treg priming. Injecting rats with rabbit nephrotoxic serum significantly up-regulated IL-10 expression (Figure 3F) (*p* < 0.05), and PsL-EGFmAb treatment further increased IL-10 expression compared with the NTN group (*p* < 0.05). There was no significant difference in TGF-β expression among the three groups (Figure 3G). Together, these results suggest that PsL-EGFmAb treatment in the rat NTN model enhanced Treg function, and that IL-10 expression might be involved.

### PsL-EGFmAb inhibits human DC maturation *in vitro*

**Figure 4:**
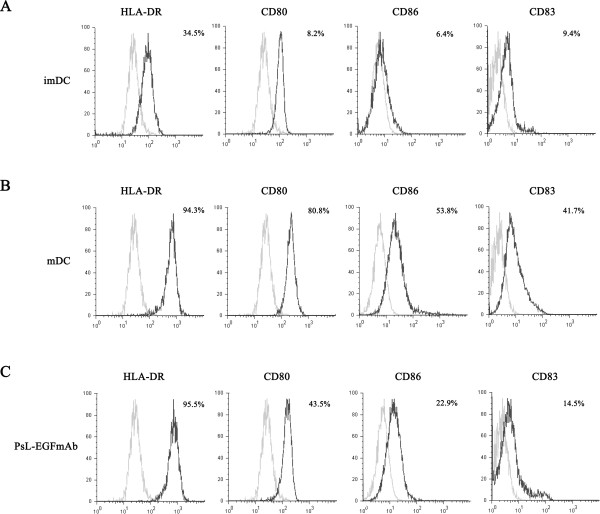
**PsL-EGFmAb inhibits human DC maturation *in vitro*. **Human CD14^+ ^monocytes from healthy donors were isolated using the MACS method and cultured in RPMI 1640 complete medium in the presence of 50 ng/ml human GM-CSF and 20 ng/ml human IL-4 for five days to produce imDCs. mDCs were induced by further treatment with 50 ng/ml TNF-α. In PsL-EGFmAb group, 10 μg/ml PsL-EGFmAb was also added before TNF-α treatment. Expression of HLA-DR, CD80, CD83 and CD86 on imDCs, mDCs, or PsL-EGFmAb-treated DCs was detected by flow cytometry. **A**, imDCs. **B**, mDCs. **C**, PsL-EGFmAb-treated DCs (black line). Appropriate isotype antibodies were used as controls (gray line). Data were representative of at least three independent experiments.

The *in vivo* data above suggested that alleviation of inflammation resulting from PsL-EGFmAb treatment was associated with a reversed Th1/Th2 cytokine profile, and was potentially the result of Treg up-regulation. DCs determine differentiation of Th0 cells to Th1 or Th2 phenotype. DCs at different maturation stages have different roles in T cell differentiation [32]. To further probe the potential mechanism by which PsL-EGFmAb treatment affects T cell functions, we used an *in vitro* system and investigated whether PsL-EGFmAb could affect DC maturation. As shown in Figure 4, A-C, flow cytometric analysis revealed that PsL-EGFmAb-treated DCs exhibited lower expression of CD80, CD86 and CD83 compared with TNF-α-induced mature DCs (mDCs). There was no difference in human leukocyte antigen (HLA)-DR expression between DCs with or without PsL-EGFmAb treatment (Figure 4, A-C).

### PsL-EGFmAb treatment promotes Treg priming mediated by DCs *in vitro*

**Figure 5:**
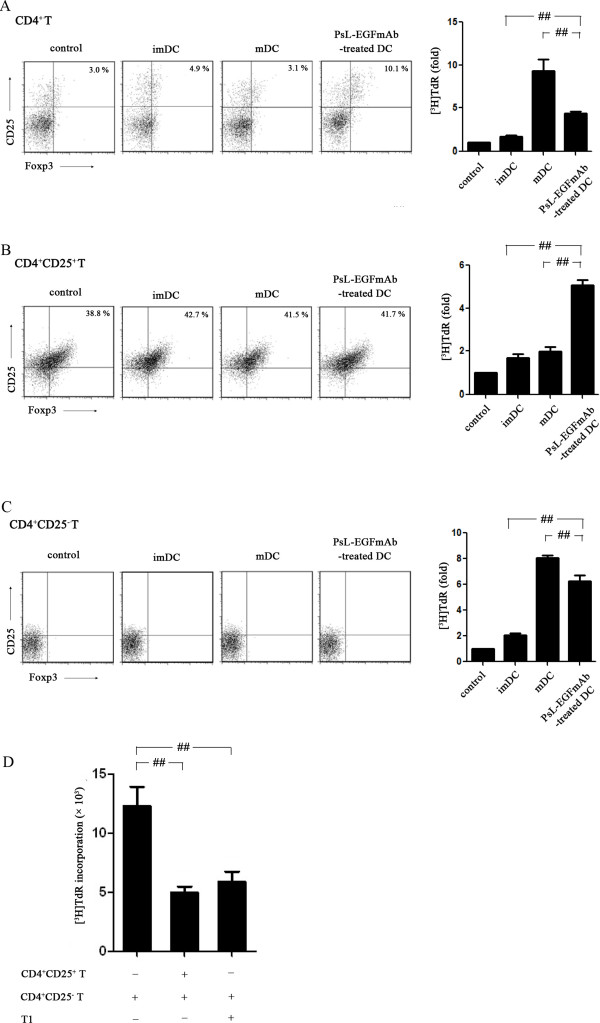
**Allogeneic mixed T cell proliferation assays. **imDCs, mDCs or PsL-EGFmAb-treated mDCs were co-cultured with freshly isolated human CD4^+^, CD4^+^CD25^+ ^and CD4^+^CD25^– ^T cells, respectively, then proliferation assays were performed and the results are shown as fold increases in [^3^H]TdR incorporation. Flow cytometric analysis was also performed to detect CD4^+^CD25^+^Foxp3^+ ^Tregs. **A**, DCs co-cultured with CD4^+ ^T cells. **B**, DCs co-cultured with CD4^+^CD25^+ ^T cells. **C**, DCs co-cultured with CD4^+^CD25^– ^T cells. **D**, A suppression assay was performed to evaluate the suppressive function of CD4^+^CD25^+ ^T cells using the same method. Tregs were either co-cultured with PsL-EGFmAb-treated mDCs (T1) or freshly isolated from health adults. The mean ± SD of four independent experiments is shown. ^#^*p* < 0.05, ^##^*p* < 0.01.

To further investigate the effect of PsL-EGFmAb on DC-mediated T cell priming, CD4^+^ T cells, CD4^+^CD25^+^ Tregs and CD4^+^CD25^-^ T cells were freshly isolated from human peripheral blood mononuclear cells (PBMCs) of healthy adults using MACS. Isolated T cells were co-cultured with imDCs, mDCs or TNF-α plus PsL-EGFmAb-treated DCs. Allogeneic mixed cell proliferation assays revealed that imDCs slightly increased the proliferation of all three types of T cells. In contrast, mDCs significantly increased proliferation of both CD4^+^ T cells and CD4^+^CD25^–^ effector T cells, but failed to increase CD4^+^CD25^+^ Treg proliferation compared with imDCs. Interestingly, PsL-EGFmAb-treated DCs displayed totally different characteristics to mDCs. PsL-EGFmAb-treated DCs significantly promoted CD4^+^CD25^+^ Treg proliferation and inhibited any further increase in CD4^+^ T cell and CD4^+^CD25^–^ effector T cell proliferation (*p* < 0.05) (Figure 5, A-C). Flow cytometric analysis revealed that PsL-EGFmAb-treated DCs also increased the percentage of CD4^+^CD25^+^Foxp3^+^ T cells after co-culture with CD4^+^ T cells (Figure 5A). CD4^+^CD25^+^Foxp3^+^ T cells were almost undetectable when DCs were co-cultured with CD4^+^CD25^-^ effector T cells (Figure 5C).

Next, we investigated whether proliferating CD4^+^CD25^+^ Tregs after co-culture with PsL-EGFmAb-treated DCs (T1 cells) could exhibit the same suppressive function as freshly isolated CD4^+^CD25^+^ Treg cells. Both T1 cells and freshly isolated CD4^+^CD25^+^ Treg cells significantly inhibited CD4^+^CD25^–^ effector T cell proliferation (*p* < 0.05) determined by suppression assay, indicating T1 cells and freshly isolated CD4^+^CD25^+^ Treg cells exhibited the same suppressive function (Figure 5D).

### IL-10 secreted by PsL-EGFmAb-treated DCs contributes to induction of Tregs

**Figure 6:**
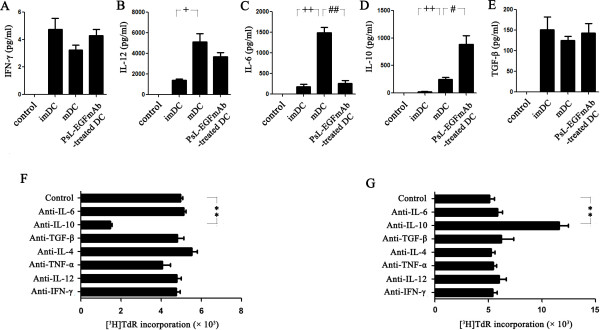
**Assays to analyze the cytokines involved in DC maturation. A**-**E**, ELISA assay for IFN-γ (**A**), IL-12 (**B**), IL-6 (**C**), IL-10 (**D**) and TGF-β (**E**) using the culture supernatants of imDCs, mDCs (TNF-α-treated DCs) and TNF-α with PsL-EGFmAb-treated DCs *in vitro*. **F**, Neutralization assays. PsL-EGFmAb-treated DCs were co-cultured with freshly isolated CD4^+^CD25^+ ^Tregs only (control) or in the presence of one of the following neutralizing anti-human antibodies *in vitro*: anti-IL-6, anti-IL-10, anti-TGF-β, anti-IL-4, anti-TNF-α, anti-IL-12 or anti-IFN-γ mAbs. The isotype-matched controls were also used. Allogeneic mixed T cell proliferation assays were performed as above. The results are shown as fold increases in [^3^H]TdR incorporation. **G**, Neutralization assays. CD4^+^CD25^+ ^T cells were co-cultured with PsL-EGFmAb-treated mDCs (T1 cell), then mixed with CD4^+^CD25^- ^effector T cells. Suppression assays were performed in the presence of the neutralizing antibodies mentioned above or with medium only (control). The mean ± SD of three independent experiments is shown. *p < 0.05, **p < 0.01 *vs.* control; ^#^*p* < 0.05, ^##^*p* < 0.01 *vs.* mDC group; ^+^*p* < 0.05, ^++^*p* < 0.01 *vs. *imDC group.

To investigate whether cytokines secreted by DCs were involved in the priming of Tregs, an enzyme-linked immunosorbent assay (ELISA) assay and neutralizing assay were performed. First we detected cytokines secreted by DCs subjected to different treatments. All DCs, despite differences in treatments, secreted low levels of IFN-γ (Figure 6A) and moderate levels of TGF-β (Figure 6E). There were no significant differences among these groups (*p* > 0.05). mDCs secreted much higher levels of IL-12 (Figure 6B) and IL-6 (Figure 6C) compared with imDCs (*p* < 0.05). However, PsL-EGFmAb treatment dramatically reduced IL-6 secretion (*p* < 0.05) but not IL-12. Although mDCs also secreted IL-10 compared with imDCs (*p* < 0.05), treatment of mDCs with PsL-EGFmAb significantly increased IL-10 secretion (Figure 6D) (*p* < 0.05). These results suggested that changes in cytokine production following PsL-EGFmAb treatment might be involved in Treg cell priming.

Next we used neutralizing antibodies to block the action of cytokines secreted by DCs to determine which cytokines were involved in Treg cell priming. Freshly isolated CD4^+^CD25^+^ Treg cells were co-cultured with PsL-EGFmAb-treated DCs in the presence of different neutralizing antibodies. CD4^+^CD25^+^ Treg cell proliferation was significantly inhibited in the presence of anti-IL-10 neutralizing antibody, whereas anti-IL-6, anti-TGF-β, anti-IL-4, anti-TNF-α, anti-IL-12 and anti-IFN-γ mAbs failed to inhibit cell proliferation, indicating that IL-10 was the key cytokine for Treg cell priming (Figure 6F).

A suppression assay confirmed these results. CD4^+^CD25^+^ T cells were co-cultured with PsL-EGFmAb-treated DCs for two days before freshly isolated CD4^+^CD25^-^ T cells were added in the presence of different neutralizing antibodies. Compared to the isotype IgG-treated group, co-cultured CD4^+^CD25^+^ T cells failed to inhibit CD4^+^CD25^-^ T cell proliferation in the presence of anti-IL-10 neutralizing antibody, whereas anti-IL-6, anti-TGF-β, anti-IL-4, anti-TNF-α, anti-IL-12 and anti-IFN-γ mAbs had no significant effect on cell proliferation (Figure 6G), indicating that IL-10 was involved in supporting the suppressive function of Tregs.

### PsL-EGFmAb targets DCs through DC-SIGN

**Figure 7:**
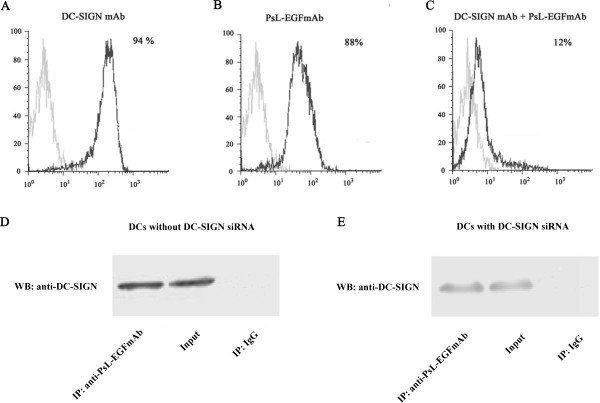
**PsL-EGFmAb signals to DCs through DC-SIGN. A**-**C**, We generated human monocyte-derived DCs by incubating MACS-isolated CD14^+ ^monocytes from healthy donor blood with GM-CSF and IL-4 for 5 days (imDCs). Flow cytometry detected DC-SIGN expression on imDCs using fluorescence-labeled DC-SIGN mAb (**A**) and PsL-EGFmAb (**B**) respectively. **C**, imDCs pre-incubated with anti-DC-SIGN goat antiserum were also included to prevent binding of PsL-EGFmAb to DCs before staining with PsL-EGFmAb. **D**, Western blotting analysis of DC-SIGN in different samples: imDC lysates immunoprecipitated using PsL-EGFmAb-coated beads (left), imDC lysates without immunoprecipitation (middle), imDC lysates immunoprecipitated using mouse IgG-coated beads (right). Experiments were performed in triplicate and the data from one representative experiment is shown. **E**, Western blotting analysis of DC-SIGN from different samples: The lysates of imDCs with DC-SIGN siRNA were immunoprecipitated using PsL-EGFmAb-coated beads (left), the lysates of imDCs with DC-SIGN siRNA without immunoprecipitation (middle), the lysates of imDCs with DC-SIGN siRNA immunoprecipitated using mouse IgG-coated beads (right).

Our data indicate that PsL-EGFmAb treatment could affect DC maturation and function, subsequently affecting Treg priming and function. Next, we investigated how PsL-EGFmAb signaled to DCs. We measured DC-SIGN expression on imDCs by flow cytometry using fluorescence-labeled DC-SIGN mAb and PsL-EGFmAb respectively. We also pre-incubated imDCs with anti-DC-SIGN goat antiserum to prevent binding of PsL-EGFmAb to DCs before staining with fluorescence labeled-PsL-EGFmAb. A similar percentage of positive cells were detected with both mAbs (Figure 7, A-C). Pre-incubation of DCs with anti-DC-SIGN goat antiserum to block DC-SIGN on DCs before detection, dramatically reduced the number of positive cells, indicating that PsL-EGFmAb likely binds to DC-SIGN on DCs.

To confirm this, immunoprecipitation was performed. DCs with or without DC-SIGN siRNA were lysed, and DC lysates immunoprecipitated with PsL-EGFmAb-coated beads. DC-SIGN was detected in immunoprecipitated DC lysates, similar to the positive control. This further supported the interaction between PsL-EGFmAb and DC-SIGN (Figure 7D, E).

## Discussion

NTN is an animal model of human crescentic GN, characterized by glomerular crescent formation, tubulointerstitial inflammation, impaired renal function and proteinuria [1,2,33]. Several studies demonstrated that a Th1-dominated nephritogenic immune response is responsible for the formation of crescents [1,2]. Recently, the protective role of Tregs in GN has been demonstrated [34]. It is well established that DCs play a critical role in the development of tubulointerstitial inflammation by modulating Th0 cells to Th1, Th2 or Th17 polarization [35], or limiting inflammation by promoting Tregs [36].

In the present study, we demonstrated that treatment with PsL-EGFmAb led to a reversal of the Th1-dominated cytokine mRNA expression profile and attenuation of renal lesions in a rat model of NTN. This was accompanied by improvement of renal function indicating that the protective effect likely involved the inhibition of DC maturation, and subsequent T cell differentiation. DCs have an important role in peripheral tolerance by various mechanisms, including activation of Tregs, induction of T cell anergy and skewed Th1/Th2 differentiation [37,38]. Increasing evidence suggests that DC functions are associated with maturation status [39,40]. Fully matured DCs are efficient activators of T cells, while imDCs have been implicated in anergy induction [41]. An intermediate stage of DC maturation was recently described, where DCs express relatively high levels of MHC class-II and co-stimulatory molecules, but do not secrete proinflammatory cytokines [42]. Previously we showed that treatment of DCs with PsL-EGFmAb, an anti-lectin-EGF domain monoclonal antibody originally developed against P-selectin, led to suppression of DC maturation and inhibition of CD4^+^ T cell proliferation *in vitro*[24]. In the present study we further demonstrated the suppression of DC maturation and T cell stimulation after PsL-EGFmAb treatment *ex vivo*, and interestingly observed an upregulation of Foxp3 and IL-10 expression in renal tissues from rat NTN. Together, treatment of PsL-EGFmAb inhibited DC maturation, which could in turn induce Treg differentiation and regulate Th1/Th2 bias.

To further characterize the effect of PsL-EGFmAb on DCs, we used human DCs that comprehensively reflected DC maturation and functions. Our results suggested that treating DCs with PsL-EGFmAb could drive DCs to an intermediate maturation stage assessed by expression of intermediate levels of co-stimulatory molecules. DCs at this maturation stage may be capable of inducing immune tolerance.

Promotion of Tregs is one mechanism by which DCs regulate immune tolerance. We therefore investigated whether PsL-EGFmAb-treated DCs could promote CD4^+^CD25^+^ Treg development. PsL-EGFmAb-treated DCs suppressed CD4^+^ T cell proliferation by stimulating the expansion of CD4^+^CD25^+^ Tregs. This raised the question of whether PsL-EGFmAb-treated DCs promoted CD25^+^ Treg proliferation or induced CD25^+^ Treg differentiation from CD25^–^ T cells. To address this question, we sorted CD4^+^CD25^–^ T cells and cultured them with PsL-EGFmAb-treated DCs. PsL-EGFmAb-treated DCs failed to differentiate CD4^+^CD25^–^ cells into CD4^+^CD25^+^ Tregs, but stimulated the expansion of primary CD4^+^CD25^+^ Tregs. Furthermore, the expanded CD4^+^CD25^+^ Tregs displayed the same ability to suppress T cell function as freshly isolated CD4^+^CD25^+^ Tregs.

IL-10 and TGF-β are immunoregulatory cytokines which facilitate the generation of murine and human Tregs [32]. Chronic activation of murine and human CD4^+^ T cells in the presence of IL-10 results in the generation of Tregs [43]. When polarized, these cells can inhibit *in vitro* and *in vivo* alloresponses [43]. In the present study, the addition of neutralizing anti-IL-10 mAb significantly inhibited CD4^+^CD25^+^ T cell proliferation induced by PsL-EGFmAb-treated DCs, and attenuated their suppressive function on CD4^+^CD25^-^ T cells. Antibodies against other cytokines had no effect, indicating a critical role of IL-10 in the promotion of Tregs by PsL-EGFmAb-treated DCs. Conversely, the above phenomenon was not seen with neutralizing anti-TGF-β mAbs.

According to the *in vivo* and *in vitro* data, we speculated that PsL-EGFmAb treatment could inhibit DC maturation in renal tissue or draining lymph nodes, to induce the generation of tolerogenic DCs, which can inhibit Th1/Th2 polarization and promote Treg proliferation by IL-10. These events together could lead to the alleviation of inflammation in renal tissue.

Next we determined which molecule on DCs interacted with PsL-EGFmAb. As PsL-EGFmAb affected DC maturation and function, this indicated that it might interact directly with DCs. As DC-SIGN has a similar lectin domain to P-selectin in its molecular structure, it may interact with PsL-EGFmAb. To test this, we compared the binding of PsL-EGFmAb and DC-SIGN mAb to DCs by flow cytometry, and found that both mAbs showed similar levels of binding. Furthermore, DCs, in which DC-SIGN was blocked by anti-DC-SIGN goat antiserum, led to dramatically reduced binding of PsL-EGFmAb. This was also supported by results from the immunoprecipitation study using PsL-EGFmAb-labeled micro beads which clearly showed that PsL-EGFmAb bound to DC-SIGN. Together, our data clearly demonstrate that PsL-EGFmAb binds to DC-SIGN on imDCs, although it can also bind to molecules containing a lectin domain. Besides DC-SIGN, PsL-EGFmAb might interact with other molecules possessing lectin or EGF domains on DCs, contributing to the immune regulation of DCs. All above are required further studies to analyze such functions.

## Conclusions

Our findings revealed a potential regulatory effect of PsL-EGFmAb, an anti-lectin-EGF domain monoclonal antibody originally developed against P-selectin, on immune responses including anti-inflammatory effects and regulation of immune reaction through DC-SIGN expressed by DCs. PsL-EGFmAb-treated DCs display an immature profile, capable of promoting CD4^+^CD25^+^ Treg expansion and inhibiting CD4^+^ effector T cell proliferation *in vitro*. The administration of PsL-EGFmAb to a rat NTN model, attenuated renal immune lesions accompanied by a skewed Th1/Th2 cytokine profile and up-regulation of Treg-related Foxp3 expression, which may have resulted from the generation of tolerogenic DCs by PsL-EGFmAb treatment. Our results suggest that PsL-EGFmAb could be a potential therapeutic treatment for human kidney diseases.

## Abbreviations

DCs: Dendritic cells; DC-SIGN: DC-specific intercellular adhesion molecule-3-grabbing non-integrin; Treg: Regulatory T cell; EGF: Epidermal growth factor; GN: Glomerulonephritis; iDCs: Immature DCs; NTN: Nephrotoxic nephritis; BUN: Blood urea nitrogen; Ccr: Creatinine clearance; GFR: Glomerular filtration rate; PAS: Periodic Acid Schiff; FBS: Fetal bovine serum; IFN: Interferon; TNF: Tumor necrosis factor; mDCs: Mature DCs; HLA: Human leukocyte antigen; PBMCs: Peripheral blood mononuclear cells; ELISA: Enzyme-linked immunosorbent assay.

## Competing interests

All the authors declared no competing interests.

## Authors’ contributions

TZ and CDX conceived the study design, participated in its design and in the acquisition of data. MCC, JW and CMM carried out the experiments, participated in the acquisition of data, analysis and interpretation, drafted the manuscript. PL have been involved in analyzing the data and provided critical advice. JMR have been involved in revising figures. JCZ and XL helped to draft and revise the manuscript. All authors read and approved the final manuscript.
